# Soft but Not Too Soft—How a Rigid Tube Expands without Breaking

**DOI:** 10.1128/mBio.00501-21

**Published:** 2021-06-15

**Authors:** Valentin Wernet, Satur Herrero, Reinhard Fischer

**Affiliations:** a Karlsruhe Institute of Technology (KIT), Karlsruhe, Germany

**Keywords:** *Aspergillus nidulans*, *Neurospora*, fungi, polar growth

## Abstract

Fungi grow by apical extension of their hyphae. The continuous growth requires constant delivery of vesicles, which fuse with the membrane and secrete cell wall biosynthesis enzymes. The growth mechanism requires the fungal cytoskeleton and turgor pressure. In a recent study by Fukuda et al. (mBio 12:e03196-20, 2021, https://doi.org/10.1128/mBio.03196-20), hyphal growth was studied in microfluidic devices with channels smaller than the hyphal diameter. The authors discovered that fast-growing fungi like Neurospora crassa enter the channels, but hyphal tips become fragile and rupture frequently, whereas slower-growing fungi like Aspergillus nidulans adapt their hyphal diameter and grow without problems through the channels. This study suggests two different growth mechanisms and a tradeoff between hyphal plasticity and growth speed.

## COMMENTARY

Polarity is a common theme in biology. Bona fide examples are the pollen tubes and root hairs in plants, protonema cells in mosses, neurons in animals, and hyphae in filamentous fungi (1). Maintenance of tube-like growth is especially challenging if an organism is surrounded by a rigid cell wall, as in the case of fungal hyphae. The cell wall consists of different glucan polymers, proteins, and a certain percentage of chitin. Essentially, fungi are living in a nutshell. Insects just remove the old skin when they need to grow, showing that growth and extension of a rigid cell wall are difficult. Well, fungi can expand the hyphae despite the rigid cell wall and even though they are pressurized by turgor like water pipes. The challenge fungi face is comparable to the extension of a polyethylene water pipe, which needs to be extended without breakage and leak of the pressurized water. The water pipe tip needs to be softened (by heat), and new polymers have to be added as the tube extends. If the tip becomes too soft, it will blow up and quickly burst. In the case of fungi, it has been known for a long time that new cell wall is synthesized exclusively at the tip and that the enzymes for cell wall biosynthesis are delivered in vesicles which fuse with the cytoplasmic membrane, thereby enlarging the membrane and secreting their enzyme content to the outside (2–4). The enzymes then synthesize new cell wall.

In a recent study by Fukuda et al. (5), the group of Norio Takeshita compared the growth of different fungi in microfluidic devices. This technique had been used before to analyze the growth of hyphae with spatial restrictions on patterned surfaces (6, 7). As a difference from previous work, the group of N. Takeshita asked what happens if the channels in such microfluidic devices are smaller than the hyphal diameter. This sounds rather artificial but actually mimics a common situation for many fungi in nature if, e.g., plant-pathogenic fungi enter a plant through the stomata or endophytic fungi grow in between plant cells, or fungal hyphae squeeze through narrow spaces in organic material. The authors found that indeed hyphae can adapt their morphology to a smaller diameter and grow through those channels with the same speed. However, the surprise came when they compared different fungi with different hyphal diameters, belonging to different fungal classes and hence having different compositions of the cell walls, or with different growth rates. They observed two different behaviors. One group of fungi continued growth after exiting the narrow channel in a normal way with tube-like hyphae. Hyphae of the other group frequently stopped growing in the channel or formed irregular, swollen structures after the channel and rarely resumed normal hyphal growth. The only parameter which correlated with the two behaviors of growth through the narrow channels was the growth speed of the hyphae. This points to two fundamentally different growth mechanisms in fungi.

**FIG 1:**
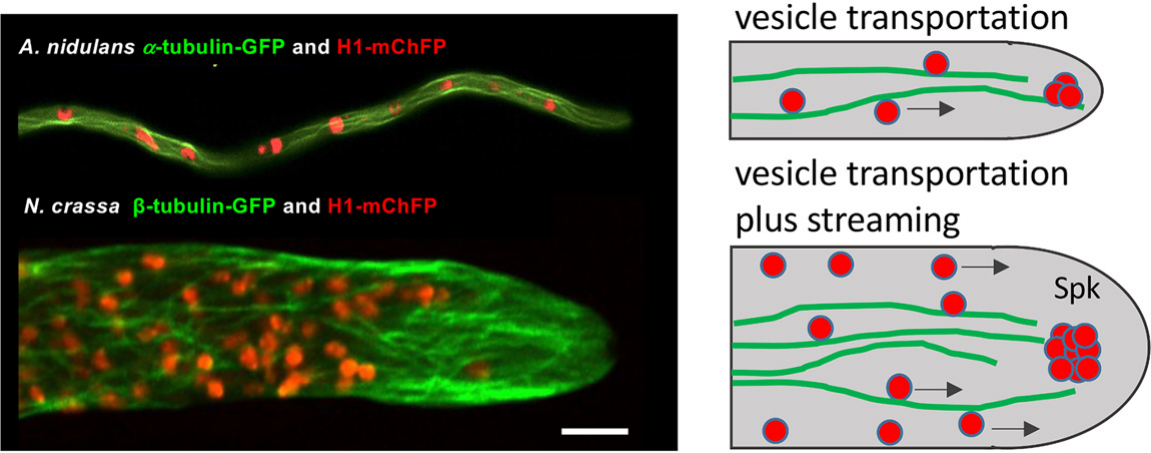
Comparison of Neurospora crassa and Aspergillus nidulans tip compartments. (Left) Visualization of the microtubule cytoskeleton and nuclei with fluorescent proteins. (Right) Vesicle transportation depends on kinesin-driven vesicle movement in A. nidulans and in N. crassa. In the fast-growing N. crassa, vesicles are also moved by cytoplasmic streaming. The Spitzenkörper (Spk) is much more pronounced in N. crassa, suggesting much larger amounts of vesicles in the apical dome. The microscopic pictures were taken from reference 1. Bar = 5 μm.

The key to understanding this interesting difference may be the arrangement of the cytoskeleton and the mode of growth in slow- and in fast-growing fungi (Fig. 1). In the slowest-growing fungus used in this study, Aspergillus nidulans, which just adapted its hyphal diameter and grew through the channels without any difficulty, the microtubule and the actin cytoskeleton are dynamic and very coordinated. Microtubules grow toward the hyphal apex and deliver so-called cell-end marker proteins to maintain polarity (1, 8). At the same time, they act as tracks for secretory vesicles, which deliver enzymes for cell wall biosynthesis, but some of them also deliver a prenylated cell-end marker protein (9). This is the receptor protein at the membrane which recruits and assembles the other components to establish the polarity site. Finally, the actin polymerase (a formin) binds to the complex and forms the polarized actin cytoskeleton emerging from the site of the cell-end markers. It should be noted that the ultimate proof for such an arrangement, the visualization of the actin cables emerging from the tip, remains to be shown. The actin filaments serve two functions: they act as tracks for the ultimate transportation of secretory vesicles, but they also capture free microtubule ends, which then slide along the actin filaments to deliver more cell-end markers to the same point (10). This causes again more actin filament formation. However, this reinforcing system is self-limiting, because the increasing number of actin filaments results in increased vesicle flow toward the cell-end marker complexes (11). The subsequent fusion of the vesicles with the cytoplasmic membrane disturbs the region where the cell-end marker complexes are clustered and causes their diffusion along the apex (12). At a new place, they may act as seeds to initiate the process again. These dynamics of the cell-end marker complexes result in local growth zones rather than a homogenous extension of the apex (13). This model is in agreement with findings in Schizosaccharomyces pombe where areas of thinner cell wall alternate with areas of thicker cell wall at the growing end (14).

In comparison, hyphae of Neurospora crassa are much wider and contain many more microtubules. The large number of microtubules allows the delivery of a large number of vesicles to the tip region. Unfortunately, cell-end marker proteins have not yet been characterized in N. crassa, and the arrangement of the actin cytoskeleton is not yet completely solved. Actin is clearly part of the Spitzenkörper, but as in A. nidulans, the exact arrangement and a connection to the cytoplasmic membrane remain to be shown. In addition to motor-driven vesicle transport along microtubules, vesicles are also transported by extensive cytoplasmic streaming, which is not obvious in A. nidulans (15–17) (Fig. 1). This probably allows moving much larger numbers of vesicles forward than by kinesin-dependent transportation along microtubules alone. However, transport may not be as easy to control and to adapt to the hyphal growth speed. The behavior in the microfluidic channel suggests that in those narrow hyphae the same number of vesicles is still produced and delivered to the apical compartment. Therefore, more vesicles may accumulate in the hyphal tip than needed. It is assumed that the hyphal cell wall is relatively malleable right after the biosynthesis and becomes more rigid through cross-link events away from the tip or that the tip is kept soft by enzymes (4). If the large number of vesicles leads to an extension of the soft cell wall area further back, it would be rather labile and, due to the turgor pressure, may break. This is exactly the observation in the study, and lowering the turgor pressure partly helped to overcome the problem. Once the hyphae exit the channel, the same overflow of vesicles could lead to a soft hyphal tip that swells and frequently explodes. Such a disturbance of the normal equilibrium between vesicle arrival and cell wall extension can be mimicked by disturbing the actin cytoskeleton in slower-growing fungi like A. nidulans (Fig. 1).

If such fundamentally different growth mechanisms exist, the question is why they evolved, or for which purpose the one or the other mechanism is adapted. The simplest explanation is the different lifestyles. N. crassa grows on organic material, and it should be advantageous to cover as much of the material as possible in a short time. Sexual spores of N. crassa are activated by heat as it occurs, e.g., after bush fires. Vegetative hyphae, but also bacteria, are probably killed at temperatures above 60°C, and hence, N. crassa resists and is one of the first organisms to grow after the fire. Therefore, it will be an advantage to grow fast before airborne competitors arrive. On the other hand, the slower growth of fungi like A. nidulans may help them to adapt better to different niches or grow in more complex substrates. The authors describe these differences as a tradeoff between morphological plasticity and velocity.

Although the results of the study are intriguing, many more interesting open questions remain, and the current publication may stimulate more of such comparative analyses. One example is the question about the solidification of the cell wall. Freshly synthesized cell wall is probably still plastic and can be extended until cross-linking enzymes, away from the tip, transform the cell wall into a rigid wall (18). Alternatively, the cell wall could actively be kept in a plastic conformation through a sophisticated and well-balanced action of synthesizing and softening enzymes (19). It will also be interesting to study if an overload of the vesicle transport machinery in slow-growing fungi can be achieved in even smaller channels.

The study is a nice example of how the ever-advancing techniques in biology and other disciplines suddenly help to solve long-standing questions which may not be solvable by traditional methods.
